# The Importance of Bacterial Culture to Food Microbiology in the Age of Genomics

**DOI:** 10.3389/fmicb.2017.00777

**Published:** 2017-05-01

**Authors:** Alexander Gill

**Affiliations:** Health Canada, Bureau of Microbial Hazards, OttawaON, Canada

**Keywords:** bacteria, culture, genomics, food, detection, characterization, subtyping

## Abstract

Culture-based and genomics methods provide different insights into the nature and behavior of bacteria. Maximizing the usefulness of both approaches requires recognizing their limitations and employing them appropriately. Genomic analysis excels at identifying bacteria and establishing the relatedness of isolates. Culture-based methods remain necessary for detection and enumeration, to determine viability, and to validate phenotype predictions made on the bias of genomic analysis. The purpose of this short paper is to discuss the application of culture-based analysis and genomics to the questions food microbiologists routinely need to ask regarding bacteria to ensure the safety of food and its economic production and distribution. To address these issues appropriate tools are required for the detection and enumeration of specific bacterial populations and the characterization of isolates for, identification, phylogenetics, and phenotype prediction.

## Introduction

Genomics, the study of the encoding, structure and function of genetic information, can be considered to have emerged as a recognized discipline with the initial publication of the eponymous journal in 1987 (McKusick and Ruddle, 1987). Initial efforts to sequence the complete genome of organisms required heroic investments of ingenuity and resources. The first prokaryote sequence, that of *Haemophilus influenzae*, was published in 1995 (Fleischmann et al., 1995) and the first eukaryote sequence, *Saccharomyces cerevisiae*, in 1996 (Goffeau et al., 1996). The first human genome was completed in 2004, following a multinational effort with an estimated cost of 3 billion dollars (Schmutz et al., 2004). The return on these investments has been techniques and tools which have dramatically lowered the time and cost of sequencing and the time and expertise required for analysis. For bacteriologists, genomic analysis is becoming a routine tool, with whole genome sequencing (WGS) available affordably, in a matter of days and with analysis supported by online platforms (Jackson et al., 2016; Kwong et al., 2016; Lindsey et al., 2016; Whiteside et al., 2016; Yoshida et al., 2016).

The foundation of bacteriology as an experimental science was the development in the 19th century of cultural techniques using solid or liquid media. Culture allowed viable bacteria to be detected and isolated, facilitated the observation of metabolic activity, and provided biomass for further analysis. As genomic analysis of bacteria becomes available as a routine tool, there is a risk of a perception developing that genomic analysis of bacteria is superior or may supersede bacterial cultural methods due to the of speed analysis and quantity of data produced. The purpose of this paper is to discuss the questions that food microbiologists routinely consider regarding bacteria, and to examine the application of cultural and genomic approaches to answering them. In food microbiology bacteria as infectious pathogens and toxin producers are a safety concern. Bacteria are also an economic concern, as their metabolic activity may enhance the economic value of foods or negatively impact it by altering sensory qualities or nutritional content.

Consideration of the strengths and limitations of cultural and genomic based methods of analysis of bacteria indicates that they provide different insights into the nature and behavior of bacteria, with the former revealing phenotypic characteristics and behavior and the later genotypic information. Neither type of information is superior to the other, but rather they should be viewed as specialized and complementary tools which are suited to answering different experimental questions.

## The Detection and Enumeration of Bacteria in Foods

The most commonly used forms of bacteriological analysis in food microbiology are detection and enumeration. The presence of specific bacteria and their concentration must be determined, to assess and control safety hazards, the potential for spoilage or to ensure correct product characteristics. The bacteria of interest to food microbiology can be divided into infectious agents, causes of foodborne intoxication, spoilage, and processing aids (**Table 1**). Metabolic activity of a bacterium may be considered as causing spoilage or as a processing aid depending upon the desirability of the changes that result.

**Table 1:** Examples of bacteria of concern to food microbiology.

Foodborne infectious agents	Foodborne intoxicants	Spoilage	Processing
*Brucella*	*Bacillus cereus*	*Acinetobacter*	Lactic Acid
*Campylobacter*	*Clostridum botulinum*	*Alcaligenes*	Bacteria
*Clostridium botulinum*	*Clostridium perfringens*	*Bacillus*	(*Lactobacillus*,
*Clostridium perfringens*	*Staphylococcus aureus*	*Brochothrix*	*Lactococcus*,
*Cronobacter*		*thermosphacta*	*Pediococcus*,
pathogenic *Escherichia coli*		*Clostridium*	*Leuconostoc*,
*Shigella*		*Cornebacterium*	*Streptococcus*)
*Salmonella enterica*		*Enterobacteriaceae*
*Yersinia enteroclitica*		*Erwinia carotovora*
*Listeria monocytogenes*		Lactic Acid Bacteria
*Mycobacterium*		*Moraxellaceae*
*Vibrio*		*Pseudomonas*
		*Shewanella*
		*putrefaciens*
		*Vibrio*


Detection of specific types of bacteria can be achieved by cultural isolation, or by indicators such as biomolecules specific to the organism (e.g., nucleic acid sequences, antigens, toxins) or products of metabolism (e.g., gas, acid, substrates with chromogenic products) (Gill et al., 2014). For enumeration cell-concentration can be estimated by partitioning the sample upon a solid surface (e.g., agar media, membrane), between liquid aliquots (e.g., most probable number) or through direct or indirect measures of biomass (e.g., optical density, Limulus amebocyte lysate assay).

Culture independent diagnostic platforms for infectious agents have been successfully commercialized in the health care sector (Caliendo et al., 2013; Zumla et al., 2014; Langley et al., 2015) and the potential for speed and automation has stimulated interest in the application of similar systems for food analysis (Anonymous, 2016a,b; Wang and Salazar, 2016). Platforms developed for health care cannot be easily adopted for use in food microbiology as analysis is significantly more challenging. Food microbiology samples are considerably more variable in type, heterogeneous in composition and the concentrations of target bacteria can be much lower. Also with the exception of stools, body fluids and tissue samples can normally be expected to contain a negligible microbiota.

The presence of non-target microbiota is a particular problem when testing for pathogens as closely related non-pathogens may result in false positives, which can have serious implications for producers. For example, the US Department of Agriculture (USDA, 2016) requires testing of raw ground beef components for shiga toxin-producing *E. coli* (STEC) which possess three traits: the virulence genes *stx* and *eae*, and six O-types considered of high risk. *E. coli* strains which possess one or two of these traits are considered of low risk and do not require the same regulatory response. The three traits, however, are not genetically linked and may be present within the sample in multiple different organisms (Delannoy et al., 2016).

Genomic technologies are considered appealing for culture-independent detection as reliability can be provided by the parallel detection of multiple genes or their transcription products. Though not currently possible, in principle, WGS using sufficiently long reads could detect and confirm the presence of the complete genome of multiple target organisms in a complex mix of DNA. In spite of accelerating advances, however, genomic technologies are not suitable for addressing two fundamental challenges in detection and enumeration; sensitivity and the determination of viability.

### The Sensitivity of Cultural Methods for Bacterial Detection

The sensitivity or limit of detection (LOD) of methods of analysis for bacterial cells is the minimum concentration of cells that can be detected. Analysis for the presence of bacteria that cause foodborne intoxication, spoilage or serve as production aids does not generally require limits of detection below 100 CFU/g or ml. Spoilage and processing bacteria do not impact the quality of a product until they exceed a significant concentration, for example spoilage of red meats by *Pseudomonads* becomes apparent above 6 log CFU/cm^2^ (Gill and Newton, 1980). Bacteria which causes intoxication need to reach relatively high concentrations in foods before significant toxin production occurs. For *C. botulinum* the threshold is 3 log CFU/g (Austin et al., 1998) and for *B. cereus* and *S. aureus* cell concentration must exceed 5 log CFU/g (FDA, 2012). However, some infectious agents have infectious doses estimated in the range of 10–100 cells (Todd et al., 2008), and the concentration of pathogen cells in outbreak associated products may be below 1 cell per 25 g (Gill and Oudit, 2015; Gill and Huszczynski, 2016). Thus, regulatory compliance testing of foods for infectious bacterial pathogens requires LODs approaching 1 cell per analytical unit, with analytical units of 10 g to 325 g depending on the specific pathogen and food (FDA, 2016; Health Canada, 2016; USDA, 2016).

Without enrichment, no existing technologies can approach this sensitivity (Wang and Salazar, 2016). Whether or not analysis is based on detection of cells or biomolecules, the target of analysis needs to be separated from the surrounding complex organic matrix, without loss of the target by adherence to the analytical apparatus. The relatively large analytical unit sizes (10 g to 325 g) make it impractical to assay anything other than a smaller aliquot of the analytic unit (0.1 to 1 ml) and many foods are composed of solids, gels and suspensions, with consequent heterogeneous distribution of bacterial cells. A method of analysis which is dependent upon the probability of the target being present in an aliquot of the analytical sample is inherently unreliable.

Cultural enrichment resolves the challenge of high sensitivity bacterial detection by amplifying the analytic target to raise the concentration and distribute it homogenously through an aqueous suspension. This ensures that detection is no longer a probabilistic process and sample handling is greatly eased. The only limitation is that the minimum time required for sample analysis is determined by the enrichment period. This will be determined by the time required for cells to begin replication (repair injury and exit lag phase) and the time required to reach the LOD of the method of analysis (growth rate). The enrichment period could be reduced by a concentration process once the analytic target is homogenously distributed in the suspension. However, the time and resources required for concentration may make this less efficient than extending the enrichment period.

### Determination of Bacterial Viability

For the bacteriological analysis of foods, it is highly desirable that the method of analysis does not confound viable cells (cells with the potential to replicate), with non-viable cells or cell debris. Foods often undergo processing steps which impact bacterial survival: the resulting population of cells may include viable cells, cells that can replicate following repair (reversibly injured) and cells which can not replicate but retain metabolic activity (irreversibly injured) (Wu, 2008). Cells of infectious agents that cannot replicate pose no threat to health. Non-replicating toxin producers only pose a threat if their concentration is already high enough to present a risk. Similarly, the presence of non-replicating cells of spoilage and processing bacteria are of no relevance to food quality.

Analytical methods which detect the presence of biomolecules, such as DNA, RNA, or proteins, cannot determine whether those biomolecules represent viable cells or not. Cell replication is a complex process, in which multiple regulatory mechanisms must coordinate the synthesis and localization of a vast array of structural and functional molecules (Reyes-Lamothe et al., 2012; Murray and Koh, 2014; Murray, 2016). The failure of the cell to complete any essential function can stall cell growth and division. Confirmation of the presence of any single essential cell component does not exclude other deficiencies that would inhibit cell replication. Thus, viability can only be determined by two methods, determination of the presence and functionality of all the molecules required, or simply waiting for the cell to exit lag phase and allowing replication to occur.

Assessment of viability may be further complicated by the potential for vegetative bacterial cells, including some foodborne pathogens, to enter into an alternate physiological state, viable-but-nonculturable (VBNC). The VBNC state can be triggered by a variety of physiochemical stresses, with cells ceasing replication but continuing metabolic activity (Pinto et al., 2015). Experimentally distinguishing VNBC states from injury is experimentally complicated, but since by definition VNBC cells can resume replication following exposure to an appropriate resuscitation stimulus (Pinto et al., 2015) identifying the correct stimulus for resuscitation rather than abandoning culture appears a more productive response.

## Charactering Bacteria

Bacterial isolates are characterized to confirm identity, to establish relationships between isolates and to understand the behavior (phenotype) of the bacterium. Though a variety of cultural and molecular methods for characterization are available genomic analysis is superseding them. Genomics may have clear superiority over other approaches in identifying and subtyping isolates, but its application to predict phenotype is much less reliable.

### Identification and Subtyping

Genomic analysis to identify isolates to the genus or species level by 16S rRNA sequence and the detection of specific gene markers by polymerase chain reaction based methods is more reliable and has greater discrimination than phenotypic methods, particularly for identification below the species level (Pace, 2009; Yilmaz et al., 2014). WGS analysis is superseding other approaches, as though complex computational analysis is required, a single wet lab process can provide information on the presence of multiple gene markers and phylogenetic relationship. Additionally, sequence data can be retrospectively analyzed for additional markers or potential relationships (Franz et al., 2016; Ronholm et al., 2016).

Establishing phylogenetic relationships for bacterial isolates below the species level can link isolates from clinical, food and environmental samples, for outbreak identification and source tracking (Fu and Li, 2014). Single polynucleotide polymorphism (SNP) analysis of WGS data can provide unprecedented discrimination between isolates (Holt et al., 2008; Chin et al., 2011). Methods for the prediction from WGS data of established genetic subtyping such as pulsed field gel electrophoresis, multilocus sequence typing and multiple-locus variable number tandem repeat analysis are being developed, but the accuracy can be compromised by short read lengths (Kwong et al., 2016; Yoshida et al., 2016). Whether subtyping of isolates is by SNP or alternatives, the only limitation on the relating isolates is the comprehensiveness of WGS and accompanying metadata available. For example, WGS data has been used to deduce the geographical origin of isolates (Weedmark et al., 2015; Hoffmann et al., 2016).

It should be noted that the application of WGS data for isolate identification, subtyping and source tracking in the context of public health, regulatory, and commercial decision making is very recent and standards for analysis and interpretation have yet to be established. Reported results are dependent upon the sequencing platform used (length of reads, error rate, genome coverage), and the data analysis pipeline (Pettengill et al., 2014; Wang et al., 2015). Data interpretation may be significantly affected by choices such as nucleotide identification algorithms, assembly method (*de novo* or reference guided) and whether the shared or core genome of isolates is compared. The need to address these issues by establishing analytical standards is recognized, but there will be a transition period until a consensus on standards emerges (Franz et al., 2016; Ronholm et al., 2016).

### Predicting Bacterial Phenotype

The complementary nature of genomic and phenotypic data is apparent when attempting to understand and predict bacterial behavior. There is wide range of bacterial behavior of interest to the food microbiologist. These include the potential for survival and replication during food production and distribution, spoilage potential, and the hazard potential of pathogens. Genomic analysis allows researchers to rapidly detect known genes, putative genes, and other defined features of the genome. However, relating genotype to phenotype with accuracy is highly challenging. Many phenotypic characteristics are the product of multiple genes and their regulatory systems. Current knowledge of any but a handful of biological regulatory systems is far from perfect. The same phenotype may result from multiple mechanisms with differing genotypes (Wilson, 2014). The phenotype may also be dependent upon interactions with other organisms (Sanchez-Vizuete et al., 2015; Chanos and Mygind, 2016). Genetically homogenous cells may be phenotypically heterogeneous, as observed in persister cells and biofilms (Grote et al., 2015; Van Acker and Coenye, 2016; Verstraeten et al., 2016). Epigenetic inheritance, DNA methylation (Adhikari and Curtis, 2016) and small RNAs (Houri-Zeevi and Rechavi, 2016) have been identified as playing a role in determining bacterial phenotype, but the mechanisms are not well understood. The significance of other potential epigenetic mechanisms such as prions (Pallarès et al., 2015), self-sustaining metabolic loops, and structural templating of membranes in bacteria is unknown (Jablonka and Lamb, 2005).

The challenges and the opportunities presented by predicting phenotype from genotype are illustrated by the prediction of antimicrobial resistance (AMR) from WGS data. Many WGS analysis platforms provide output of AMR associated genes, but the utility of this information is questionable. A 2016 review of the potential for AMR prediction by WGS conducted for the European Committee on Antimicrobial Susceptibility Testing concluded that, for the purposes of informing clinical decisions, “The published evidence for using WGS as a tool to infer antimicrobial susceptibility accurately is currently either poor or non-existent” (Ellington et al., 2016). Though prediction accuracy may be limited, the ability to rapidly screen large populations of strains for a potential phenotype is still useful. Knowles et al. (2016) used WGS data to determine whether a specific STEC strain possessed AMR genes that were relatively uncommon among *E. coli* and determined the presence of trimethoprim resistance genes. Trimethoprim resistance was confirmed experimentally and the addition of trimethoprim to enrichment broth was demonstrated to aid isolation (Knowles et al., 2016). In an outbreak investigation, this approach could be used in the analysis of foods for strains previously isolated from patients.

The purpose of discussing the limitations of genomics is not to imply that the application of genomics to answer these questions is inappropriate. When complemented with phenotypic data and studies of physiological mechanisms, genomic data is a powerful tool to improve our understanding, but the challenges in relating genomic data to phenotype must be recognized. Decision making related to food safety or food processing should not be made solely on the basis of genomic data, but needs to be supported by phenotypic data, which in turn require culture. When grounded in phenotypic data, genomic data has the potential to enhance culture methods (Knowles et al., 2016) or to develop culture method for previously unculturable organisms (Renesto et al., 2003). As databases of WGS data expand, genomic analysis can be used to rapidly screen large populations for strains that potentially possess desired phenotypes, or to select experimental strains that are representative of a larger population.

## Conclusion

Genomic technologies are tools with enormous potential for increasing our understanding of bacteria and solving practical problems in food microbiology, but like any tools the benefits and costs are dependent upon how we choose to employ them. Medical microbiology is faced with a set of unnecessary challenges due to the trend of abandoning cultural isolation for culture-independent diagnostic testing. At the clinical level this presents difficulties distinguishing viable from non-viable organisms and in data interpretation when multiple organisms are present. At the public health level this results in the inability to collect epidemiological data such as, subtype and AMR, and prohibits further characterization and research (Janda and Abbott, 2014; Huang et al., 2016). Food microbiologists should learn from this example and consider how to maximize the benefits without losing the advantages of alternate technologies. Genomic analysis is becoming the standard method for the identification and phylogenetics of bacteria, but culture remains necessary to achieve the required sensitivity of detection and enumeration and to determine viability. Just as crucially, culture is needed to provide the isolates with which to conduct experiments to test hypothesizes generated from genomic data. When phenotype is predicted from genomic data we are creating a model of a biological system and the great value of such models as noted by Jeremy Gunawardena (2014) is to reveal the limitations of our understanding.

## Author Contributions

The author confirms being the sole contributor to this work and approved it for publication.

## Conflict of Interest Statement

The author declares that the research was conducted in the absence of any commercial or financial relationships that could be construed as a potential conflict of interest.
